# Balancing and tight coupling: an approach to determine dynamic mechanisms of biological brain networks

**DOI:** 10.1186/1471-2202-15-S1-A3

**Published:** 2014-07-21

**Authors:** Frances K Skinner

**Affiliations:** 1 Krembil Discovery Tower, Toronto Western Hospital, 60 Leonard Street, 7KD411 Toronto, ON, Canada M5T 2S8

## 

Without a doubt, it is an extremely challenging endeavour to understand how our brains work. Oscillatory output, as produced by brain networks, has been shown to be important for brain functioning. Due to the high degree of sophistication and technical expertise required in experimentation, modeling, computation and analyses, it is clear that to move forth in our understanding, open and interactive collaborations between several individuals and disciplines are required.

In this talk, I will discuss our developing approach to determine essential features and mechanisms for the generation of rhythmic, population output in microcircuits of the hippocampus. Due to its importance in learning and memory, as well as its association with pathological conditions, the hippocampus is a heavily studied brain structure. Furthermore, evidence is accumulating that pathological states are associated with particular changes in normal rhythmic activities. Through collaborative efforts, we have developed and are developing cellular and network models with tight experimental linkages. We are using them to identify critical cellular and synaptic aspects of dynamic mechanisms that can be examined in biological microcircuits. Overall, we aim to use our models to determine dynamic mechanisms used by biological microcircuits (from which one could consider building macrocircuits) and to use them to gain insight into disease mechanisms.

